# Closed-form spin-relativistic corrections from the Dirac equation enabling a modified Schrödinger solver

**DOI:** 10.1038/s41598-025-29243-4

**Published:** 2025-12-21

**Authors:** Mário B. Amaro, Nazeef, Camille J. Dussech, Chong Qi

**Affiliations:** 1 Department of Physics, KTH Royal Institute of Technology, Alba Nova Centre, S-106 91 Stockholm, Sweden; 2 Department of Physics, Stockholm University, Alba Nova Centre, S-106 91 Stockholm, Sweden; 3 Department of Physics, Birla Institute of Technology and Sciences Pilani, Hyderabad Campus, Hyderabad, India; 4 Université de Bordeaux, Talence Cedex, France; 5 Academy of Romanian Scientists, Ilfov Street, no.3, 050044 Bucharest, Romania

**Keywords:** Mathematics and computing, Physics

## Abstract

We revisit the non-relativistic limit of the Dirac equation in finite scalar and vector potentials and derive a Schrödinger-like equation that retains leading spin–relativistic corrections in closed form. For general central potentials, we cast the radial equation into a quadratic eigenvalue problem (QEP) using a finite-difference discretization method and develop an open-source solver to address it. We study Coulomb, harmonic oscillator, Woods–Saxon, and Yukawa potentials. We further obtain first-order energy and wavefunction corrections for the three-dimensional isotropic harmonic oscillator and Coulomb potentials via perturbation theory. This framework provides a practical bridge between non-relativistic and fully relativistic treatments, enabling accurate quantification of relativistic effects without the computational cost of full four-component calculations.

## Introduction

The Dirac equation, introduced nearly 100 years ago in 1928, represents the most successful attempt to incorporate the effects of special relativity into quantum mechanics in describing the behaviour of spin-1/2 particles. One fundamental departure from the standard Schrödinger equation is that the Dirac equation describes particles with 4-component spinors (two bi-spinors) $\Psi =(\phi _l,\phi _s)^T$ instead of scalar wavefunctions $\psi$. Nevertheless, the product $\Psi ^{\dagger }\Psi$ is positive-definite, and is still associated with the particle probability density^1^. That can be compared to the Born’s postulate of quantum mechanics that the wave function product $\rho (r,t)=\psi ^{\dagger }\psi =|\psi (r,t)|^2$ defines the probability density of finding a particle at position *r* and time *t*. It may be interesting to mention that, in the de Broglie–Bohm interpretation of quantum mechanics (Bohmian mechanics), the link between the probability density and the wave function appears naturally as a result of the dynamic consequences of the quantum equilibrium hypothesis.

It may be interesting to mention that the Dirac equation for a free particle reduces to the Schrödinger equation in the non-relativistic limit where the 4-component spinors acquire the approximate form

$$\begin{aligned} \Psi = \begin{pmatrix}\phi _l \\ \phi _s\end{pmatrix} e^{-imc^2t/\hbar } \end{aligned}$$

where both $\phi _l$ and $\phi _s$ are two-component spinors. The spinor $\psi _l$ has the explicit form

$$\begin{aligned} \phi _l(\textbf{r},t)=\xi e^{i(\textbf{p}\cdot \textbf{r}-E't)/\hbar }, \end{aligned}$$

with

$$\begin{aligned} \phi _s \approx \frac{\varvec{\sigma }\cdot \textbf{p}}{2mc}\phi _l \end{aligned}$$

where $E'=E-mc^2$ and $\xi$ is a constant normalized Pauli spinor. The two-component spinors $\phi _l$ and $\phi _s$ are often respectively referred to as the large and small components of the Dirac wave functions. $\phi _l$ approaches the non-relativistic wave function $\psi$ as energy decreases while $\phi _s$ vanishes. In a recent work by Wilczek and Yu^2^, it is argued that the usual identification of $\psi ^*\psi$ as the probability density is conceptually unsatisfactory as the product $\Psi ^{\dagger }\Psi$ contains a relativistic correction in the form

1 $$\begin{aligned} \rho = \Psi ^{\dagger }\Psi \approx \psi ^*\psi +\frac{1}{4m^2}(\nabla \psi ^*\cdot \nabla \psi ) \end{aligned}$$

A fundamental consequence is that the density thus defined will never vanish, except at infinity, as one can not have the wave function and its derivative approach zero at the same time.

The studying of the relativistic effects on electron probability density has played an important role in many field of fundamental physics studies including quantum field theory, subatomic physics and nuclear astrophysics. The Dirac equation incorporates relativity inherently. One often studies the relativistic effect by comparing the solutions of the Dirac and Schrödinger equations. There has been long-standing interest in not only solving the Dirac equation but also deriving its non-relativistic limits (see, for examples, Ref^3–5^.). But it should be clarified that not all “relativistic corrections” arising from the Dirac equation are purely kinematic; some originate from its spinor structure, which introduces new spin physics beyond relativistic kinematics. The purpose of this paper is to reinvestigate the non-relativistic limit of the Dirac equation for particles in a finite potential and to explore the effect of these spin-relativistic correction terms to the Schrödinger equation that arise in this limit, leading to a good bridge between non-relativistic and fully relativistic treatments that can be employed whenever moderate but non-negligible relativistic effects must be quantified, without having to resort to a full relativistic treatment, which introduces computational complexity. We will show that a closed-form Schrödinger-like equation can be derived from taking the non-relativistic expansion of the Dirac equation. The modified equation contains non-linear terms but can be solved using the finite–difference method, with which the system is transformed into a quadratic eigenvalue problem (QEP). Unlike usual perturbation studies, the solving of the QEP matrix gives us consistently the non-relativistic corrections to the energies, wave functions, as well as the probabilistic density.

There is a wide literature of semi-relativistic approximation schemes to the Dirac equation to decouple large and small components and deliver a two or one component Hamiltonian with relativistic effects retained at a controlled cost. Foldy-Wouthuysen and the related Direct Perturbation Theory perform unitary block-diagonalizations order-by-order yielding Pauli-like effective Hamiltonians. Douglas-Kroll-Hess perturbation theory refines this with a sequence of transformations constructed to treat scalar potentials more gently and to converge more smoothly at higher orders. The methods discussed so far are perturbative methods with tunable order and broad reliability, but by no means the only options. By contrast, Barysz-Sadlej-Snijders method constructs an energy-independent decoupling that is exact at the one-electron level, and then adds corrections for two-electron terms, leading to a compact, near-exact two-component model. Other lighter and more approximate methods exist, such as the zeroth-order regular approximation (ZORA), which eliminates the small component via a low-order regularization of the kinetic-balance ratio. This last method is cheap at the cost of being the most approximate. Many other methods exist in the literature. A good overview of these methods can be found in works such as the textbooks by Dyall and Fægri^6^ and Reiher and Wolf^7^. The approach explored in this work retains the leading spin-relativistic operators explicitly in a Schrödinger-like equation and collects momentum-dependent pieces into an energy and potential-dependent effective mass, solvable via a QEP. It may also be interesting to mention that in the area of precision molecular physics, there are interest in high-precision evaluation of leading-order relativistic and QED corrections which has been explored with parts-per-million accuracy using explicitly correlated basis functions and regularization techniques^8–10^.

## Derivation from Dirac Hamiltonian

We start with a general Dirac Hamiltonian with scalar potential S and vector potential V:

2 $$\begin{aligned}&H \Psi = [\vec {\alpha }\cdot \vec {p} + \beta (m + S) + V] \Psi , \end{aligned}$$

3 $$\begin{aligned}&[E - \vec {\alpha }\cdot \vec {p} - \beta (m +S) - V] \Psi = 0 , \end{aligned}$$

where *m* is the mass of the particle, *E* is the energy and

$$\begin{aligned} \vec {\alpha } = \begin{pmatrix} 0 & \vec {\sigma } \\ \vec {\sigma } & 0 \end{pmatrix}, \beta = \begin{pmatrix} 1 & 0 \\ 0 & -1 \end{pmatrix}. \end{aligned}$$

It can be written in a simpler matrix form as

4 $$\begin{aligned} \begin{pmatrix} E - (m + S) - V & -\vec {\sigma }\cdot \vec {p} \\ -\vec {\sigma }\cdot \vec {p} & E + (m + S) - V \\ \end{pmatrix} \begin{pmatrix} \phi _l \\ \phi _s \end{pmatrix} = 0, \end{aligned}$$

which gives us two coupled equations

5 $$\begin{aligned} (E - m - \Sigma ) \phi _l - \vec {\sigma }\cdot \vec {p} \phi _s = 0 \end{aligned}$$

6 $$\begin{aligned} -\vec {\sigma } \cdot \vec {p} \phi _l + ( E + m - \Delta ) \phi _s = 0 \end{aligned}$$

where we have taken

$$\begin{aligned} \Sigma = V + S, \\ \Delta = V - S. \\ \end{aligned}$$

The equations can be written in second derivative form as

7 $$\begin{aligned} (E- m - \Sigma ) \phi _l = [\vec {\sigma }\cdot \vec {p} ( E + m - \Delta )^{-1} \vec {\sigma }\cdot \vec {p}] \phi _l. \end{aligned}$$

Taking the non-relativistic limit:

$$\begin{aligned} E \rightarrow {E' + m}, \end{aligned}$$

the above equation can be rewritten as

8 $$\begin{aligned} (E' - \Sigma )\phi _l = \vec {\sigma }\cdot \vec {p} ( E' + 2m - \Delta )^{-1} \vec {\sigma }\cdot \vec {p} \phi _l. \end{aligned}$$

Using Taylor series expansion, the equation can be approximated as

$$\begin{aligned} (E' - \Sigma )\phi _l =\frac{1}{2m} \vec {\sigma }\cdot \vec {p} \left( 1 - \frac{E' - \Delta }{2m}\right) \vec {\sigma }\cdot \vec {p} \phi _l. \end{aligned}$$

From now on, we will replace $E'$ with *E* for simplicity as we focus on the non-relativistic regime. The above equation can be further rewritten as

9 $$\begin{aligned} E \phi _l = \left[ \frac{p^2}{2m} + \Sigma - \frac{E p^2}{4m^2} + \frac{\vec {\sigma }\cdot \vec {p} \Delta \vec {\sigma }\cdot \vec {p}}{4m^2} \right] \phi _l. \end{aligned}$$

By expanding the fourth term on right side in the form

10 $$\begin{aligned} \begin{aligned} \sigma \cdot \vec {p} \, \Delta \, \sigma \cdot \vec {p} =&\sum _{i,j} \sigma _i p_i \Delta \sigma _j p_j = \sum _{i,j} (\delta _{ij} + i \epsilon _{ijk} \sigma _k) p_i \Delta p_j \\ =&\sum _i p_i \Delta p_i + i \sum _{i,j,k} \epsilon _{ijk} \sigma _k p_i \Delta p_j, \end{aligned} \end{aligned}$$

and by replacing

$$\begin{aligned} \vec {p} = -i \nabla , \end{aligned}$$

we have

11 $$\begin{aligned} \sum _i \partial _i \Delta \, \partial _i \psi = \sum _i \left( (\partial _i \Delta )(\partial _i \psi ) + \Delta \, \partial _i^2 \psi \right) . \end{aligned}$$

So, as an operator:

12 $$\begin{aligned} & \sum _i \partial _i \Delta \, \partial _i = (\nabla \Delta ) \cdot \nabla + \Delta \nabla ^2 \end{aligned}$$

13 $$\begin{aligned} & \sum _i p_i \Delta p_i = -\left( (\nabla \Delta ) \cdot \nabla + \Delta \nabla ^2 \right) \end{aligned}$$

The so-called Darwin term is often considered in non-relativistic studies^11,12^. Considering that $\vec {p}$ and $\Delta$ may not commute, the first term up to the second order term is given by

14 $$\begin{aligned} \sum _i p_i \Delta p_i = \sum _i (\Delta p_i p_i + [p_i, \Delta ] p_i + \frac{1}{2} [p_i, [\Delta , p_i]]), \end{aligned}$$

where

$$\begin{aligned} [p_i, \Delta ] = -i\partial _i \Delta \end{aligned}$$

and the double commutator is

$$\begin{aligned} [p_i, [\Delta , p_i]] = \nabla ^2 \Delta . \end{aligned}$$

For the second term:

15 $$\begin{aligned} i \sum _{i,j,k} \epsilon _{ijk} \sigma _k p_i \Delta p_j = i \, \vec {\sigma } \cdot (\vec {p} \Delta \times \vec {p}) = \vec {\sigma } \cdot (\nabla \Delta \times \vec {p}). \end{aligned}$$

Combining both terms above, we get the full expansion:

16 $$\begin{aligned} \vec {\sigma }\cdot \vec {p}\Delta \vec {\sigma }\cdot \vec {p} = \vec {\sigma }\cdot (\nabla \Delta \times \vec {p}) - i \nabla \Delta \cdot \vec {p} + \Delta p^2 + \frac{1}{2} \nabla ^2 \Delta . \end{aligned}$$

We reach the final form of the Schrödinger equation-like non relativistic limit of the Dirac equation

17 $$\begin{aligned} \begin{aligned} E \phi _l =&\left[ \frac{p^2}{2m} + \Sigma - \frac{E p^2}{4m^2} + \frac{\vec {\sigma }.\nabla \Delta \times \vec {p}}{4m^2} \right. \\ &\left. - \frac{i \nabla \Delta \cdot \vec {p}}{4m^2} + \frac{\Delta p^2}{4m^2} + \frac{\nabla ^2 \Delta }{8m^2} \right] \phi _l , \end{aligned} \end{aligned}$$

which includes, as expected, the standard Schrödinger equation terms as well as energy- and potential-dependent relativistic correction terms.

The Darwin term appears in some Schrödinger-equivalent potential derived from the Dirac equation including for examples Refs^13–15^.. The Darwin term is generally small compared to the central and spin-orbit terms. It may be interesting to point out that the Darwin ends up as a constant correction for a harmonic oscillator, which can therefore be neglected, and a surface correction for a Woods-Saxon potential. For the Coulomb potential, it is known to acquire a Dirac function form $\delta (r)$ which affects only the *s* wave at the origin.

Now we would like to re-express the equation in explicit second derivative form. We will be now writing the equation explicitly. We assume spherical symmetry and that the potentials only dependent on the radius. In spherical coordinates, we have for any function *f*

$$\begin{aligned} \nabla f = \frac{1}{r} \frac{d f}{d r} \vec {r}. \end{aligned}$$

Therefore, we have for the second correction term

18 $$\begin{aligned} \frac{\vec {\sigma \cdot \nabla \Delta \times \vec {p}}}{4m^2} = \frac{1}{4m^2}\frac{1}{r}\frac{d \Delta }{d r} \vec {\sigma }\cdot [\vec {r} \times \vec {p} ] \end{aligned}$$

which represents the spin-orbit coupling, and can be written in the more known form as

$$\begin{aligned} \vec {\sigma }\cdot [\vec {r} \times \vec {p}] = \vec {\sigma }\cdot \vec {L}. \end{aligned}$$

We can define an operator $\hat{k}$, as often done in the solving of the Dirac equation, in the form

$$\begin{aligned} \hat{k} = \beta (\vec {\sigma }\cdot \vec {L} + 1). \end{aligned}$$

The eigenvalues of $\hat{k}$ are such that, $k = -(l+1)$, if $j = l+ \frac{1}{2}$ and $k = +l$ if $j = l -\frac{1}{2}$. Given that operator $\hat{k}$ acts on spin spherical harmonics $\Omega _{mk}(\theta ,\phi )$, as follows:

$$\begin{aligned} \hat{k}\Omega _{mk} = -k\Omega _{mk} \end{aligned}$$

The fourth correction term for the upper spinor is:

19 $$\begin{aligned} \frac{\vec {\sigma \cdot \nabla \Delta \times \vec {p}}}{4m^2} = \frac{1}{4m^2}\frac{1}{r}\frac{d \Delta }{d r} (-k - 1). \end{aligned}$$

The fifth term can be rewritten as

20 $$\begin{aligned} - \frac{i \nabla \Delta \cdot \vec {p}}{4m^2} = \frac{1}{4m^2} \frac{d \Delta }{d r} \frac{\partial }{\partial r}, \end{aligned}$$

where we applied the following property:

$$\begin{aligned} (\nabla f)\cdot \nabla = \frac{d f}{d r} \frac{\partial }{\partial r}. \end{aligned}$$

The sixth term is:

$$\begin{aligned} \frac{\Delta p^2}{4m^2} = -\frac{\Delta \nabla ^2}{4m^2} \end{aligned}$$

The non-relativistic limit of the Dirac equation in spherical polar coordinates:

21 $$\begin{aligned} \begin{aligned} E \phi _l =&\left[ -\frac{\nabla ^2}{2m} + \Sigma + \frac{E \nabla ^2}{4m^2} -\frac{1}{4m^2}\frac{1}{r}\frac{d \Delta }{d r} (k + 1) \right. \\ &\left. + \frac{1}{4m^2} \frac{d \Delta }{d r} \frac{\partial }{\partial r} -\frac{\Delta \nabla ^2}{4m^2} + \frac{\nabla ^2 \Delta }{8m^2} \right] \phi _l \end{aligned} \end{aligned}$$

where it may be useful to remind that $\phi _l$ contains two components and that for standard $\nabla ^2$ operator one has

22 $$\begin{aligned} \begin{aligned} \nabla ^2 =&\frac{1}{r^2} \frac{\partial }{\partial r} \left( r^2 \frac{\partial }{\partial r} \right) + \\ &+ \frac{1}{r^2 \sin \theta } \frac{\partial }{\partial \theta } \left( \sin \theta \frac{\partial }{\partial \theta } \right) + \frac{1}{r^2 \sin ^2 \theta } \frac{\partial ^2 }{\partial \phi ^2}. \end{aligned} \end{aligned}$$

We can thus separate the form of 21 into radial (*R*(*r*)) and angular ($\Omega _{mk}(\theta ,\phi )$) components. Collecting all the terms with the radial component, multiplying them by $2r^2$

23 $$\begin{aligned} \begin{aligned}&2r^2(E-\Sigma )R+\frac{r}{2m^2}\frac{d\Delta }{dr}(k+1)R + \\ &+\frac{1}{m} \left[ 1 -\frac{E - \Delta }{2m}\right] \frac{d}{dr}\left( r^2\frac{dR}{dr}\right) - \frac{r^2}{2m^2}\frac{d\Delta }{dr}\frac{dR}{dr} - \frac{r^2\nabla ^2 \Delta R}{4m^2} . \end{aligned} \end{aligned}$$

The third term above is exactly the inverse of the effective mass given in the following section, in Equation 60, multiplying the equation by $\bar{m}$ and equating the radial terms to the separation constant

24 $$\begin{aligned} \begin{aligned}&2\bar{m}r^2(E-\Sigma )R+\frac{\bar{m}r}{2m^2}\frac{d\Delta }{dr}(k+1)R +\frac{d}{dr}\left( r^2\frac{dR}{dr}\right) \\ &-\frac{\bar{m}r^2}{2m^2}\frac{d\Delta }{dr}\frac{dR}{dr} - \frac{\bar{m}r^2\nabla ^2 \Delta R}{4m^2} - l(l+1)R=0. \end{aligned} \end{aligned}$$

Dividing the whole by $r^2$ we arrive at

25 $$\begin{aligned} \begin{aligned}&2\bar{m}(E-\Sigma )R+\frac{\bar{m}}{2m^2r}\frac{d\Delta }{dr}(k+1)R +\frac{1}{r^2}\frac{d}{dr}\left( r^2\frac{dR}{dr}\right) \\ &-\frac{\bar{m}}{2m^2}\frac{d\Delta }{dr}\frac{dR}{dr}- \frac{\bar{m}\nabla ^2 \Delta R}{4m^2}-\frac{l(l+1)}{r^2}R=0. \end{aligned} \end{aligned}$$

We have considered both potentials *S* and *V* in our derivation. It may be interesting to mention that both the concepts of spin and pseudospin symmetries are often discussed in relativistic quantum mechanics. Spin symmetry arises in the Dirac equation when the difference between the vector and scalar potentials, $\Delta (r) = V(r) - S(r)$, is approximately constant, $\Delta (r) \approx \text {const}$, while pseudospin symmetry appears when the sum of the potentials, $\Sigma (r) = V(r) + S(r)$, is nearly constant, $\Sigma (r) \approx \text {const}$^16^. These symmetries are typically understood as emerging from the decoupling of the large and small components of the Dirac spinor, leading to degenerate doublets in the spectrum.

In our formulation, we focus on the modified Schrödinger equation obtained in the non-relativistic limit which retains the spin–orbit and Darwin terms explicitly in closed analytical form, without requiring the restrictive assumptions that are usually imposed to achieve exact spin or pseudospin symmetry. This approach therefore allows for a broad class of potentials and provides a flexible framework to study relativistic corrections and fine-structure effects in systems where spin and pseudospin symmetry may be only approximate or partially realized (see, for example, Ref^17^. and references therein).

### Analytical solutions

The modified Schödinger equation distilled from the Dirac equation(for a purely radial potential), can be expressed as below second-order differential equation

26 $$\begin{aligned} \begin{aligned} \frac{d^2R}{dr^2}&+ \left[ \frac{2}{r} -a_o \frac{dV}{dr} \right] \frac{dR}{dr} + \left[ 2m(E-V)+ \right. \\ &\left. +\frac{a_o}{r} \frac{dV}{dr}(k+1) -\frac{a_o}{2} \nabla ^2 V -\frac{l(l+1)}{r^2} \right] R =0 \end{aligned} \end{aligned}$$

where,

27 $$\begin{aligned} a_o = \frac{\bar{m}}{2m^2}. \end{aligned}$$

For $R(r) = \phi (r) u(r)$, the function $\phi (r)$ is chosen such that the first-order term disappears from the differential equation. In the following discussion, we treat the effective mass $\bar{m}$ as a constant and use it to construct $\phi$ as

28 $$\begin{aligned} \phi (r) = \frac{1}{r} e^{\frac{a_o V}{2}}, \end{aligned}$$

substituting this in Equation 26 we get,

29 $$\begin{aligned} \begin{aligned} \frac{d^2u}{dr^2} + \left[ \frac{a_oV^{'}}{r} (k \right.&\left. +1) - \left( \frac{a_o V^{'}}{2} \right) ^2 +\right. \\ &\left. +2m(E-V) - \frac{l(l+1)}{r^2} \right] u = 0. \end{aligned} \end{aligned}$$

The above equation is valid for a general potential dependent on r except for a Coulomb-like potential with inverse r dependence. The differential equation for such a potential is given below

30 $$\begin{aligned} \begin{aligned} \frac{d^2u}{dr^2} + \left[ \frac{a_o V^{''}}{2} \right.&\left. + \frac{a_oV^{'}}{r} (k+2) - \left( \frac{a_o V^{'}}{2} \right) ^2+ \right. \\ &\left. +2m(E-V) -\frac{a_o}{2} \nabla ^2 V - \frac{l(l+1)}{r^2} \right] u = 0. \end{aligned} \end{aligned}$$

Both the above equations are in Schrödinger-like equation form

31 $$\begin{aligned} \left( \frac{d^2}{dr^2}+ V_{\text {eff}} \right) u = 0, \end{aligned}$$

depending on the chosen potential, such equations may admit exact or quasi-exact solutions. We have demonstrated how analytical solutions for the Harmonic oscillator and the Coulomb potentials can be derived in the following subsections along with their Perturbative analysis in the next section.

#### Harmonic oscillator

When affected by a Harmonic oscillator potential such as $m \omega ^2 r^2/2$ Equation 29 becomes,

32 $$\begin{aligned} \begin{aligned}&\frac{d^2u}{dr} + \left[ 2mE + a_om\omega ^2(k+1)+ \right. \\&\left. - \left( \frac{a_o^2 \omega ^2}{4}+1 \right) m^2 \omega ^2r^2 -\frac{l(l+1)}{r^2}\right] u= 0. \end{aligned} \end{aligned}$$

We introduce $\kappa$ to write the coefficient of $r^2$ in a succinct form,

33 $$\begin{aligned} \kappa =\left( \frac{a_o^2 \omega ^2}{4}+1 \right) m^2 \omega ^2 . \end{aligned}$$

Expressing derivatives in *r* as primed quantities, Equation 32 becomes

34 $$\begin{aligned} \begin{aligned} u'' + \left[ 2mE + a_om\omega ^2(k+1)- \kappa r^2 -\frac{l(l+1)}{r^2}\right] u= 0. \end{aligned} \end{aligned}$$

Now, observing the asymptotic behaviour of this equation for $r \rightarrow \infty$, Equation 34 reduces to

35 $$\begin{aligned} u'' = \kappa u. \end{aligned}$$

The form of the equation suggests that the solution exhibits Gaussian-like behaviour in the limit of large *r*,

36 $$\begin{aligned} u = c_1 e^{-\frac{\sqrt{\kappa } r^2}{2}}. \end{aligned}$$

Similarly, at the limit *r* is very small, Equation 34 reduces to

37 $$\begin{aligned} u'' = \frac{l(l+1)}{r^2} u. \end{aligned}$$

In this limit, the solution takes the form

38 $$\begin{aligned} u = c_2r^{l+1}. \end{aligned}$$

Combining the results from asymptotic analysis gives a general solution of the form

39 $$\begin{aligned} u = r^{l+1}e^{\frac{-\sqrt{\kappa }r^2}{2}} \sigma (r), \end{aligned}$$

expressing $\sigma (r)$ as a power series

40 $$\begin{aligned} \begin{aligned}&\sigma (r) = \sum _{j=0}^\infty a_j r^j, \\ &\end{aligned} \end{aligned}$$

and substituting the expression for $\sigma (r)$ in Equation 34, we get a recurrence relationship,

$$\begin{aligned} \begin{aligned} a_{j+2} = -\frac{a_om\omega ^2(k+1) + 2mE -\sqrt{\kappa }(2l+3+2j)}{(j+2)(2l+3+j)}a_j. \end{aligned} \end{aligned}$$

The above recurrence relation enables us to find the expression for energy levels

41 $$\begin{aligned} \begin{aligned} E_n = \frac{\sqrt{\kappa }(2l+3+2n) - a_om\omega ^2(k+1)}{2m}. \end{aligned} \end{aligned}$$

The recurrence equation is exactly that of an associated Laguerre polynomial, hence $\sigma (r)$ can be succinctly expressed as

42 $$\begin{aligned} \sigma (r) = L^{l+\frac{1}{2}}_n (\sqrt{\kappa }x^2). \end{aligned}$$

The exact solution for *R*(*r*) can be expressed by multiplying $\phi (r)$ (from Equation 28) and *u*(*r*),

43 $$\begin{aligned} R(r) = r^l e^{\frac{a_om\omega ^2-2\sqrt{\kappa }}{4}r^2} L^{l+\frac{1}{2}}_n (\sqrt{\kappa }x^2) . \end{aligned}$$

#### Coulomb potential

When potential $V = -\alpha /r$, where $\alpha$ is the fine-structure constant times the atomic number *Z*, is substituted in Equation 30,the equation for all points except $r=0$, takes a Doubly-Confluent Heun equation form,

44 $$\begin{aligned} \begin{aligned} u'' + \left[ -\frac{a_o^2\alpha ^2}{4r^4} + \frac{a_o \alpha (k+1)}{r^3} \right.&\left. - \frac{l(l+1)}{r^2}+ \right. \\&\left. + \frac{2m\alpha }{r} + 2mE\right] u = 0. \end{aligned} \end{aligned}$$

The above equation is also of the Kratzer-like potential Schrödinger equation ($Hu=0$) form

45 $$\begin{aligned} \begin{aligned} H = \left[ \frac{d^2}{dr^2} + \frac{\lambda }{r^4} + \frac{\mu }{r^3} + \frac{\nu }{r^2} + \frac{\xi }{r} +\tau \right] , \end{aligned} \end{aligned}$$

where the corresponding coefficients are

46 $$\begin{aligned} \begin{aligned} \lambda&= -\frac{a_0^2 \alpha ^2}{4}, \quad \quad&\mu&= a_0 \alpha (k+1), \\ \nu&= -l(l+1) ,&\xi&= 2m\alpha , \\ \tau&= 2mE. \end{aligned} \end{aligned}$$

A broad class of exactly and quasi-exactly solvable models has been established through algebraic and analytical methods^18–20^. There were also studies on general conditions for obtaining polynomial solutions of second-order differential equations^21^ and solvable Schrödinger potentials using confluent Heun functions^22^. Refs^23,24^. applied these principles to relativistic wave equations including the Dirac and Duffin–Kemmer–Petiau formulations which suggest that solvable structures persist in quantum-corrected and spin-one settings. To derive the quasi-exact solution, we introduce the following transformation:

47 $$\begin{aligned} u = r^{1-\eta } e^{-\sqrt{-\tau }r -\frac{\sqrt{-\lambda }}{r}} \Omega (r), \end{aligned}$$

where,

48 $$\begin{aligned} \eta = \frac{\mu }{2\sqrt{-\lambda }}, \end{aligned}$$

such that $1-\eta> 0$ and $\tau ,\lambda <0$. After substituting values of $\lambda$ and $\mu$ in Equation 48, we get $\eta =(k+1)$, for the condition $1-\eta>0$ to always be true, *k* takes the value $-(l+1)$. The Hamiltonian can now be expressed as $\bar{H} \Omega =0$,

49 $$\begin{aligned} \begin{aligned} \bar{H}= r^2\frac{d^2}{dr^2}-2(\sqrt{-\tau }r^2-(1-\eta )r-\sqrt{-\lambda })\frac{d}{dr}-(\gamma r+\zeta ) \end{aligned} \end{aligned}$$

where,

50 $$\begin{aligned} \begin{aligned}&\gamma = 2(1-\eta )\sqrt{-\tau } -\xi , \\ &\zeta = \eta (1-\eta )+2\sqrt{\lambda \tau }-\nu .\\ \end{aligned} \end{aligned}$$

This Hamiltonian can be expressed as a quadratic combination of generators of an $\mathfrak {sl}(2)$ set

51 $$\begin{aligned} \begin{aligned} \bar{H}_{QES} = -J_n^+J_n^-&+2\sqrt{-\tau }J_n^+-2\sqrt{-\lambda }J_n^-+\\ &+(2\eta -n-2)\left( J_n^0+\frac{n}{2}\right) +\zeta , \end{aligned} \end{aligned}$$

if $\gamma$ is of the form,

52 $$\begin{aligned} \gamma = -2n\sqrt{-\tau }, \quad \quad n=0,1,2... \end{aligned}$$

The generators of the $\mathfrak sl(2)$ set are

53 $$\begin{aligned} J_n^+=r^2\frac{d}{dr}-nr, \quad J_n^0=r\frac{d}{dr}-\frac{n}{2}, \quad J_n^-=\frac{d}{dr}. \end{aligned}$$

Using the relation introduced by Equation 52, we can derive the expression for energy levels

54 $$\begin{aligned} E_n = -\frac{2m\alpha ^2}{(k-n)^2}. \end{aligned}$$

The ability to construct the Hamiltonian in terms of quadratic combinations of $\mathfrak sl(2)$ generators implies that it leaves invariant an $(n+1)$-dimensional finite vector space. Hence, $\bar{H}_{QES}$ preserves the finite-dimensional space of polynomials of degree at most *n*,

55 $$\begin{aligned} \Omega (r) = \sum _{j=0}^n c_jr^j \end{aligned}$$

Substituting the value of $\Omega$ in *u*, followed by evaluating the Hamiltonian equation, we get a three-term recurrence relation,

56 $$\begin{aligned} \begin{aligned} c_{m+1}=\frac{(\zeta +m(2\eta -m-1))c_m+2(m-n-1)\sqrt{-\tau }c_{m-1}}{2(m+1)\sqrt{-\lambda }} \end{aligned} \end{aligned}$$

with $c_{-1}=c_{n+1}=0$. Finally, the radial part of the wavefunction *R* can be expressed as product of $\phi$ and *u*,

57 $$\begin{aligned} \begin{aligned} R(r) = r^{l} \, e^{-\left( \sqrt{-2mE}\, r + \frac{a_0 \alpha }{r} \right) } \sum _{j=0}^{n} c_j \, r^{j}. \end{aligned} \end{aligned}$$

In the limit $2m>>(E-V)$, $2m>>1$, it follows that $\bar{m}= a_0 =0$. In this regime, the radial solutions given by equations 43 and 57 reduce to their corresponding solutions of the Schrödinger equation.

### Effective mass

We will refer to the non-relativistic limit of the Dirac equation with the correction terms as the modified Schrödinger equation for simplicity. The modified equation can be simplified by considering specific terms to be contributing to an effective mass:

58 $$\begin{aligned} \frac{p^2}{2\bar{m}} =&\frac{p^2}{2m} -\frac{Ep^2}{4m^2} + \frac{\Delta p^2}{4m^2}, \end{aligned}$$

59 $$\begin{aligned} \bar{m} =&\frac{m}{1- \frac{(E - \Delta )}{2m}}. \end{aligned}$$

Using again the Taylor expansion, we have:

60 $$\begin{aligned} \bar{m} \approx m \left( 1 + \frac{E - \Delta }{2m} \right) . \end{aligned}$$

### Probability density

The general Dirac Spinor for a particle in a finite potential has now the form

$$\begin{aligned} \Psi = \begin{pmatrix} \phi _l \\ \frac{1}{E + 2m - \Delta }\vec {\sigma }\cdot \vec {p} \phi _l. \end{pmatrix} \end{aligned}$$

The Dirac density probability is:

61 $$\begin{aligned} \begin{aligned} \rho =\Psi ^{\dagger } \Psi =&\phi _l^{\dagger }\phi _l +\left( \frac{1}{E + 2m - \Delta }\vec {\sigma }\cdot \vec {p} \phi _l \right) ^{\dagger }\times \\ \times&\left( \frac{1}{E + 2m - \Delta }\vec {\sigma }\cdot \vec {p} \phi _l \right) . \end{aligned} \end{aligned}$$

Taking again the non-relativistic limit:

$$\begin{aligned} E - \Delta \ll 2m, \end{aligned}$$

we then have

$$\begin{aligned} \rho = \phi _l^{\dag }\phi _l + \frac{1}{4m^2} \left( \phi _l^{\dag } p^2 \phi _l \right) . \end{aligned}$$

In our derivation above, we considered the general Dirac Hamiltonian containing both scalar *S*(*r*) and vector *V*(*r*) potentials to ensure formal completeness. For the applications discussed below, primarily atomic and electronic systems where relativistic effects are most relevant, the scalar term is either negligible or can be effectively absorbed into the mass term. Therefore, we focused on the case $S = 0$ (or equivalently $\Sigma = \Delta = V$) to emphasize the physically dominant vector potential contribution.

The derivation, however, remains fully general, and the inclusion of a finite scalar potential is straightforward by reintroducing *S*(*r*) through the definitions $\Sigma = V + S$ and $\Delta = V - S$. Such scalar contributions are mainly relevant in nuclear, hypernuclear, or quark-level systems, where Lorentz-scalar couplings modify the rest mass. Extending the present framework to those regimes would require a more elaborate treatment of the poorly constrained vector and scalar potentials.

## Perturbation corrections

### Harmonic oscillator

Despite the added complexity, it is of interest to search for analytical solutions to Equation 24, which can be done by identifying it as a Schrödinger equation with correction terms, allowing us to employ perturbation-theoretical methods to find good analytical approximations. In this section, we apply this method to find approximate analytical expressions for the useful case of the harmonic oscillator. We start by taking simply a vector potential and no scalar potential such that $\Sigma =\Delta =V(r)$, and therefore Equation 24 becomes

62 $$\begin{aligned} \begin{aligned}&2\bar{m}\,(E-V)R + \frac{\bar{m}}{2m^2\,r}\frac{dV}{dr}(k+1)R+\frac{1}{r^2}\frac{d}{dr}\Bigl (r^2\frac{dR}{dr}\Bigr )\\ &-\frac{\bar{m}}{2m^2}\frac{dV}{dr}\frac{dR}{dr}- \frac{\bar{m}\nabla ^2 V R}{4m^2}-\frac{l(l+1)}{r^2}R=0\,. \end{aligned} \end{aligned}$$

Since the $\bar{m}$ term has an explicit *E* and *V* dependence, we substitute its form back in Equation 62, together with the form of the potential $V(r)=\frac{1}{2}m\omega ^2r^2$ and arrive at

63 $$\begin{aligned} \begin{aligned} \frac{1}{r^2}\frac{d}{dr}\Bigl (r^2\frac{dR}{dr}\Bigr )+2m(E-V)R+(E-V)^2R-\\ +\frac{\omega ^2}{2}(k+1)R+\frac{\omega ^2}{4m}(E-V)(k+1)R-\frac{\omega ^2r}{2}\frac{dR}{dr}\\frac{\omega ^2\,r}{4m}(E-V)\frac{dR}{dr}-\frac{3 \omega ^2 R}{4}-\frac{3 \omega ^2}{8m}(E-V)R=\frac{l(l+1)}{r^2}R. \end{aligned} \end{aligned}$$

It is now of interest to reorganize this result into two parts. One is an unperturbed Hamiltonian $H_0$ that corresponds to the standard harmonic oscillator pieces and a perturbation Hamiltonian $H'$ that contains the extra terms. We have that

64 $$\begin{aligned} \begin{aligned} H_0 R\equiv \frac{1}{r^2}\frac{d}{dr}\Bigl (r^2\frac{dR}{dr}\Bigr )-\frac{l(l+1)}{r^2}R+\\+2m\Bigl [E-\tfrac{1}{2}\,m\omega ^2r^2\Bigr ]R, \end{aligned} \end{aligned}$$

which gives us the zeroth order energy as the usual eigenvalues of the harmonic oscillator, given by

65 $$\begin{aligned} E^{(0)}=\hbar \omega \Bigl (2n+\ell +\frac{3}{2}\Bigr ), \end{aligned}$$

with *n* and *l* the radial and angular quantum numbers, respectively. As for the perturbation, we have

66 $$\begin{aligned} \begin{aligned} H'R \equiv&(E-V)^2R+\frac{\omega ^2}{2}(k+1)R+\\ &+\frac{\omega ^2}{4m}(E-V)(k+1)R-\frac{\omega ^2r}{2}\frac{dR}{dr}+\\ &-\frac{\omega ^2r}{4m}(E-V)\frac{dR}{dr}-\frac{3 \omega ^2 R}{4}-\frac{3 \omega ^2}{8m}(E-V)R. \end{aligned} \end{aligned}$$

Working to first order in our small expansion parameter, and keeping in mind that $E-V$ is much smaller than *m*, we can make a series of observations. Firstly, we can take in terms that multiply an expectation value or that serve as correction factors the approximation

67 $$\begin{aligned} E-V\approx E^{(0)}-\frac{1}{2}m\,\omega ^2r^2. \end{aligned}$$

Secondly, we assume the terms involving *dR*/*dr* to vanish upon integration by parts under the standard boundary conditions. This allows us to, for the purpose of computing the first-order corrections, work only with

68 $$\begin{aligned} \begin{aligned} H'(r)=\Bigl (E^{(0)}-\frac{1}{2}m\omega ^2r^2\Bigr )^2 +\frac{\omega ^2}{2}(k+1) +\\+\frac{\omega ^2}{4m}(k+1)\Bigl (E^{(0)}-\frac{1}{2}m\omega ^2r^2\Bigr )\ \frac{3 \omega ^2}{4}-\frac{3 \omega ^2}{8m}\Bigl (E^{(0)}-\frac{1}{2}m\omega ^2 r^2\Bigr ). \end{aligned} \end{aligned}$$

The time-independent perturbation theory tells us that

69 $$\begin{aligned} E^{(1)}=\langle R^{(0)}|H'|R^{(0)}\rangle , \end{aligned}$$

And therefore

70 $$\begin{aligned} \begin{aligned} E^{(1)}\approx&\left\langle \Bigl (E^{(0)}-\frac{1}{2}m\omega ^2r^2\Bigr )^2\right\rangle +\frac{\omega ^2}{4}(2k-1)\langle 1\rangle +\\ &+\frac{\omega ^2}{8m}(2k-1)\left\langle E^{(0)}-\frac{1}{2}m\omega ^2r^2\right\rangle \,. \end{aligned} \end{aligned}$$

Here, a series of simplifications are possible, namely the fact that via the virial theorem for the harmonic oscillator, one finds that

71 $$\begin{aligned} \langle r^2\rangle =\frac{E^{(0)}}{m\omega ^2} \end{aligned}$$

and therefore the first term adopts the neat form

72 $$\begin{aligned} \left\langle \Bigl (E^{(0)}-\frac{1}{2}m\omega ^2r^2\Bigr )^2\right\rangle =\frac{1}{4}m^2\omega ^4\langle r^4\rangle , \end{aligned}$$

and therefore we finally find

73 $$\begin{aligned} E^{(1)} \approx \frac{1}{4} m^2\omega ^4\langle r^4\rangle + \frac{\omega ^2}{4}(2k-1) + \frac{\omega ^2}{16m}(2k-1)E^{(0)}, \end{aligned}$$

where the fourth moment $\langle r^4\rangle$ computed in the unperturbed state can usually be expressed in a closed form as a function of the quantum numbers. As for the first-order correction to the radial wavefunction, we have the standard formula

74 $$\begin{aligned} R^{(1)}_{n\ell }(r)=\sum _{n'\ne n}\frac{\langle R^{(0)}_{n'\ell }|H'|R^{(0)}_{n\ell }\rangle }{E^{(0)}_{n}-E^{(0)}_{n'}}\,R^{(0)}_{n'\ell }(r)\,, \end{aligned}$$

where $H'$ is given by the form of Equation 68. Exploiting the orthogonality of the oscillator eigenfunctions, one can deduce that only the $r^2$ and $r^4$ pieces will contribute to the wavefunction correction as the constant pieces in $H'$ would yield matrix elements proportional to $\langle R^{(0)}_{n'\ell }|1|R^{(0)}_{n\ell }\rangle$, which is vanishing for $n'\ne n$. Therefore, the first-order correction to the wavefunction is given by Equation 74 using an effective Hamiltonian

75 $$\begin{aligned} H'_{e}=\frac{1}{4}m^2\omega ^4r^4-m\omega ^2\left( E^{(0)}+\frac{\omega ^2}{16}(2k+5)\right) r^2 \end{aligned}$$

### Coulomb potential

Using the same approach as for the Harmonic Oscillator, we shall now derive the perturbation correction for the case of a Coulomb potential, given by:

76 $$\begin{aligned} V(r)&= -\frac{e^2}{4 \pi \epsilon _0 \hbar c} \frac{Z}{r}. \end{aligned}$$

This yields a perturbation of the form

77 $$\begin{aligned} H'(r)= \frac{\bar{m} \alpha }{2m^2r^3}(k+1)-\frac{\bar{m} \alpha }{2m^2r^2R}\frac{dR}{dr} - \frac{\pi \bar{m} \alpha }{m^{2}} R \delta ^{3}(r), \end{aligned}$$

where we define for the sake of compactness

78 $$\begin{aligned} \alpha = \frac{e^2Z}{4 \pi \epsilon _0 \hbar c}. \end{aligned}$$

The last term is the Darwin term, which is henceforth omitted, as we shall evaluate the energies for $l>0$ for which the Dirac delta function term will have no contribution. Using Equation 74:

79 $$\begin{aligned} \begin{aligned} E^{(1)}=\frac{+ \alpha \bar{m}}{2m^2}(k+1)&\langle R^{(0)}|\frac{1}{r^3}|R^{(0)}\rangle + \\ &- \frac{\alpha \bar{m}}{2m^2}\langle R^{(0)}|\frac{1}{r^2R}\frac{dR}{dr}|R^{(0)}\rangle . \end{aligned} \end{aligned}$$

Let us first focus on the initial bracketed term. Since

80 $$\begin{aligned} R_{nl}(r) = N e^{-r / (n a_0)} \left( \frac{r}{n a_0} \right) ^{\ell } L_{n - \ell - 1}^{2\ell + 1} \left( \frac{2r}{n a_0} \right) , \end{aligned}$$

one finds that

81 $$\begin{aligned} \langle \frac{1}{r^3}\rangle = \frac{Z^3}{n^3 a_0}\frac{1}{l(l+1/2)(l+1)}. \end{aligned}$$

For a Coulomb potential, the radial wavefunction behaves as $R(r)\propto \exp (-2r)$, which vanishes as $r\rightarrow \infty$ due to the exponential decay. Near the origin, the behavior is $R \propto r^l$ so $R(r) \Rightarrow 0$ as $r\rightarrow 0$ for all $l>0$. Let us now examine the second bracketed term:

82 $$\begin{aligned} \langle R^{(0)}|\frac{1}{r^3}|R^{(0)}\rangle = \frac{1}{2}\int _{0}^{\infty } (\frac{dR}{dr}) (R(r))^2 = 0 \end{aligned}$$

Therefore, we find the energy correction to be

83 $$\begin{aligned} E^{(1)}= \frac{Ze^2}{4\pi \epsilon _0 \hbar c}\frac{\bar{m}}{2m^2}(k+1)(\frac{Z}{n a_0})^3 \frac{1}{l(l+1/2)(l+1)} \end{aligned}$$

## Numerical implementation

In order to study the prevalence of the effects described in the previous section, a numerical implementation of Equation 24 was developed. The enforcing of the wavefunction boundary conditions is significantly simplified if we introduce a variable change $u=rR$, leading to

84 $$\begin{aligned} \begin{aligned}&\frac{d^2u}{dr^2}-\left( \frac{\bar{m}}{2m^2}\frac{d\Delta }{dr}\right) \frac{du}{dr}+\left( 2\bar{m}(E-\Sigma )\right. +\\ &+\left. \frac{\bar{m}(k+2)}{2m^2r}\frac{d\Delta }{dr} - \frac{\bar{m} \nabla ^2 \Delta }{4m^2}\right) u=\frac{l(l+1)}{r^2}u. \end{aligned} \end{aligned}$$

For $r,u\rightarrow \infty$, we can simply impose that the wavefunction vanish. The main advantage of this variable change is that, for $u\rightarrow 0$, *R*(*u*) is strictly zero, while for $r\rightarrow 0$ *R*(*r*) is simply finite, and a non-zero boundary condition would have to be enforced. As shall be seen, our numerical implementation employs the Finite Difference Method (FDM), where zero Dirichlet boundary conditions are convenient.

### Finite Difference Method (FDM)

The Finite Difference Method (FDM) is the name given to a series of techniques that rely on discretizing the parameter space in a mesh and approximate derivatives using finite differences, effectively reducing the problem to a linearized version of itself, making it significantly simpler^25^. This has the advantage of allowing the usage of highly optimized matrix operation routines to solve the problem in a time- and resource-efficient way. With a sufficiently small step between grid points, the derivatives are well-approximated by the finite differences. The discretization of the parameter space is most often done using a uniform spacing, although methods of discretization for arbitrarily spaced meshes exist as well (e.g^26^^27^^28^.). In this work, a uniform spacing was used. A typical approach would be the use of second-order accuracy central first- and second-order derivatives of *u*(*r*)

85 $$\begin{aligned} \begin{aligned}&\frac{du(r)}{dr} \approx \frac{u(r+h) - u(r-h)}{2h},\\&\frac{d^2u(r)}{dr^2} \approx \frac{u(r+h) - 2 u(r) + u(r-h)}{h^2}, \end{aligned} \end{aligned}$$

where we let *h* denote the step of the mesh. However, in order to increase the accuracy of the solver, we can increase the number of mesh points to the left and right of the point of interest that we involve in the calculation, effectively increasing the accuracy of the finite difference. Let *m* and *n* be the number of points to the left and right of *i*, $c_k$ be coefficients obtained by solving the system from the Taylor expansion and q the order of accuracy, we can derive finite differences of arbitrary order of accuracy to approximate the p-th derivative of *u*(*r*) at point *i* by expanding $u(r\pm kh)$ in a Taylor series around $r_i$ as

86 $$\begin{aligned} u^{(p)}(r_i) \;\approx \; \frac{1}{h^p}\sum _{k=-m}^{n} c_k \, u_{i+k} + \mathcal {O}(h^q). \end{aligned}$$

In this work, we opted for a five-point central difference scheme ($q=4$). This increases the accuracy significantly from the simple near-neighbor three-point scheme, and is a usual technique to avoid the well-known spurious state problem that arises in FDM approaches to the Dirac equation^29^^30^, and should also arise in its non-relativistic limit^31^.

The FDM has been extensively employed in the field, namely in numerical implementations of the Schrödinger^32^^33^^34^ and Dirac^35^^29^^30^ equations, in both time-dependent and -independent contexts. Other common numerical methods include Runge-Kutta 4^36^^37^, and specifically for the more intricate Dirac equation methods such as mapped Fourier method^38^, Green’s function method^39^^40^, evolutionary algorithms^41^ or power-series expansion^42^ among others have been employed.

### Numerical method

One immediately sees that Equation 84, equipped with the respective boundary conditions, is an eigenvalue problem with eigenfunction *u*(*r*) and respective eigen energies *E*, which can be solved after finite-difference discretization as a matrix eigenvalue problem. It should be noted, however, that the factor $\bar{m}$, defined in Equation 60, also includes an *E* term. Therefore, the term of Equation 84 where $\bar{m}$ and *E* are multiplied is effectively quadratic in *E*, meaning that we are dealing with a Quadratic Eigenvalue Problem (QEP), a special case of non-linear eigenvalue problem. For a survey of this method see e.g^43^.. Upon explicit substitution of Equation 60 onto Equation 84, we are able to write it in an eigenvalue form

87 $$\begin{aligned} (E^2 A+E B+C)u=0, \end{aligned}$$

88 $$\begin{aligned} \begin{aligned}&A= 1, \\&B=\left( 2m-\Sigma -\Delta +\frac{k+2}{4m^2r}\frac{d\Delta }{dr}-\frac{\nabla ^2\Delta }{8m^2}\right) -\frac{1}{4m^2}\frac{d\Delta }{dr}\frac{d}{dr}, \\&C=\left( -2m\Sigma +\Delta \Sigma +\frac{k+2}{2mr}\frac{d\Delta }{dr}-\frac{(k+2)\Delta }{4m^2r}\frac{d\Delta }{dr}\right. - \frac{\nabla ^2\Delta }{4m}+\\&+\frac{\Delta \nabla ^2\Delta }{8m^2}-\left. \frac{l(l+1)}{r^2}\right) -\left( \frac{1}{2m}-\frac{\Delta }{4m^2}\right) \frac{d\Delta }{dr}\frac{d}{dr} +\frac{d^2}{dr^2}.\\ \end{aligned} \end{aligned}$$

Upon discretization, the resulting linear systems can be written as matrices. Let *N* be the number of points of the mesh, then *A*, *B* and *C* will become $N\times N$ matrices. Note that, due to the boundary conditions that we enforce, we know that $u_0=u_N=0$, meaning that the first and last rows and columns of the matrices $\textbf{A}$, $\textbf{B}$ and $\textbf{C}$ can be casted away and the matrices can be reduced to their non-trivial $(N-1)\times (N-1)$ core.

The method was implemented in Python, where the standard numerical computation libraries such as NumPy^44^ or SciPy^45^ don’t feature solvers for nonlinear eigenvalue problems. Since, as aforementioned, the main reason for using the FDM is the possibility to exploit the highly optimized linear algebra algorithms featured in these packages, one can transform our current problem into a generalized eigenvalue problem, which is supported, and solve that one instead. Starting from our current form

89 $$\begin{aligned} (E^2\textbf{A}+E\textbf{B}+\textbf{C})u=0, \end{aligned}$$

and by employing an auxiliary vector $v=Eu$, we get

90 $$\begin{aligned} E(\textbf{A}v+\textbf{B}u)=-\textbf{C}u \Rightarrow \begin{pmatrix} \textbf{B} & \textbf{A} \\ \textbf{I} & \textbf{0} \end{pmatrix} U= \begin{pmatrix} -\textbf{C} \\ \textbf{I} \end{pmatrix}U, \end{aligned}$$

where $$U=\begin{pmatrix}u&v\end{pmatrix}^T$$, which can now be solved as a generalized eigenvalue problem using the routines in the aforementioned libraries. It should also be noted that, as usual in FDM implementations, the resulting matrices are sparse matrices, contributing to the numerical efficiency of the eigenvalue problem solving routines. Nonetheless, solving a quadratic eigenvalue problem is significantly more computationally demanding than the usual eigenvalue problem. The main reason for this is that the generalized linear problem that we obtained in Equation 90 increases the cost from $\mathcal {O}(n^3)$ to $\mathcal {O}((2n)^3)$. We therefore end up with a matrix with twice the size, and twice the number of eigenvalues, and hence twice the spectral work. It should also be noted that the newly obtained form is non-Hermitian, which requires the usage of more complex algorithms.

Due to the fact that we are discretizing the space using a uniform grid with constant spacing, one must be attentive to the fact that some states can end up poorly resolved at the lower-*r* end, at distances of the order of magnitude of the step size. If neglected, this can hinder the retrieval of their wavefunctions and energies of deep states such as $1s_{1/2}$. In order to ensure that the results in the following section are accurate, we wrote a second code with logarithmic spacing to cross-check that the energy levels and wavefunctions were exactly reproduced in both, ensuring that the results were robust and not resolution-dependent.

### Numerical results

**Fig. 1:**
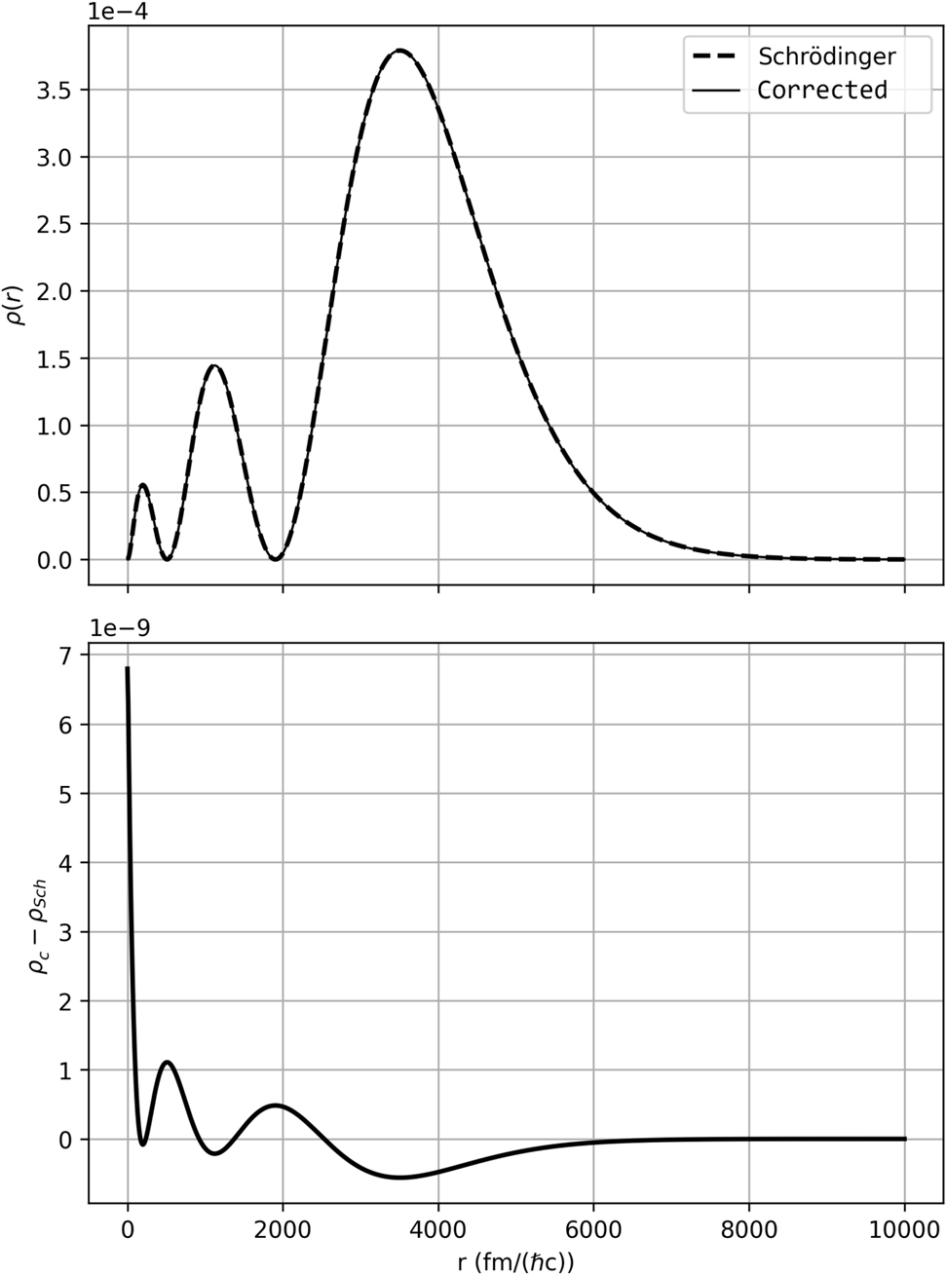
(Top) Comparison of the Hydrogen $3s_{1/2}$ state probability density solved using both the standard Schrödinger equation and the modified form described in this work; (Bottom) Magnitude of the correction, given by the difference between the corrected and standard probability densities. The effect of the correction terms is negligible, as expected.

One expects the contribution of the relativistic corrections to be extremely subtle for low energies, which was put to test as both a benchmark and sanity check using the $3s_{1/2}$ electronic state of the Hydrogen atom, with a binding energy of about $-1.5\mathrm {~eV}$. To retrieve it, a Coulomb potential was employed, with the familiar form

91 $$\begin{aligned} V(r)=-\frac{e^2}{4\pi \epsilon _0\hbar c}\frac{Z}{r}, \end{aligned}$$

with *e* the elementary charge, $\epsilon _0$ the vacuum permittivity and *Z* the number of protons in the nucleus. Note that we are using the unit $\mathrm {fm/\hbar c}$ or $\mathrm {MeV^{-1}}$ for distance. This is depicted in Fig. 1 (Top), where the standard radial Schrödinger equation solution and the numerical solution to the corrected form derived in this work are plotted against each other. They are virtually indistinguishable, as expected. Nonetheless, there is a small correction, which is easily seen in Fig. 1 (Bottom), where the standard wavefunction was subtracted from the corrected one. It is interesting to notice that the correction, although small, gives us some insights on the effect of the corrections: where the standard wavefunction vanishes, the corrected one is non-vanishing, since a contribution appears due to the $\nabla \psi ^*\cdot \nabla \psi$ term in Equation 1.

**Fig. 2:**
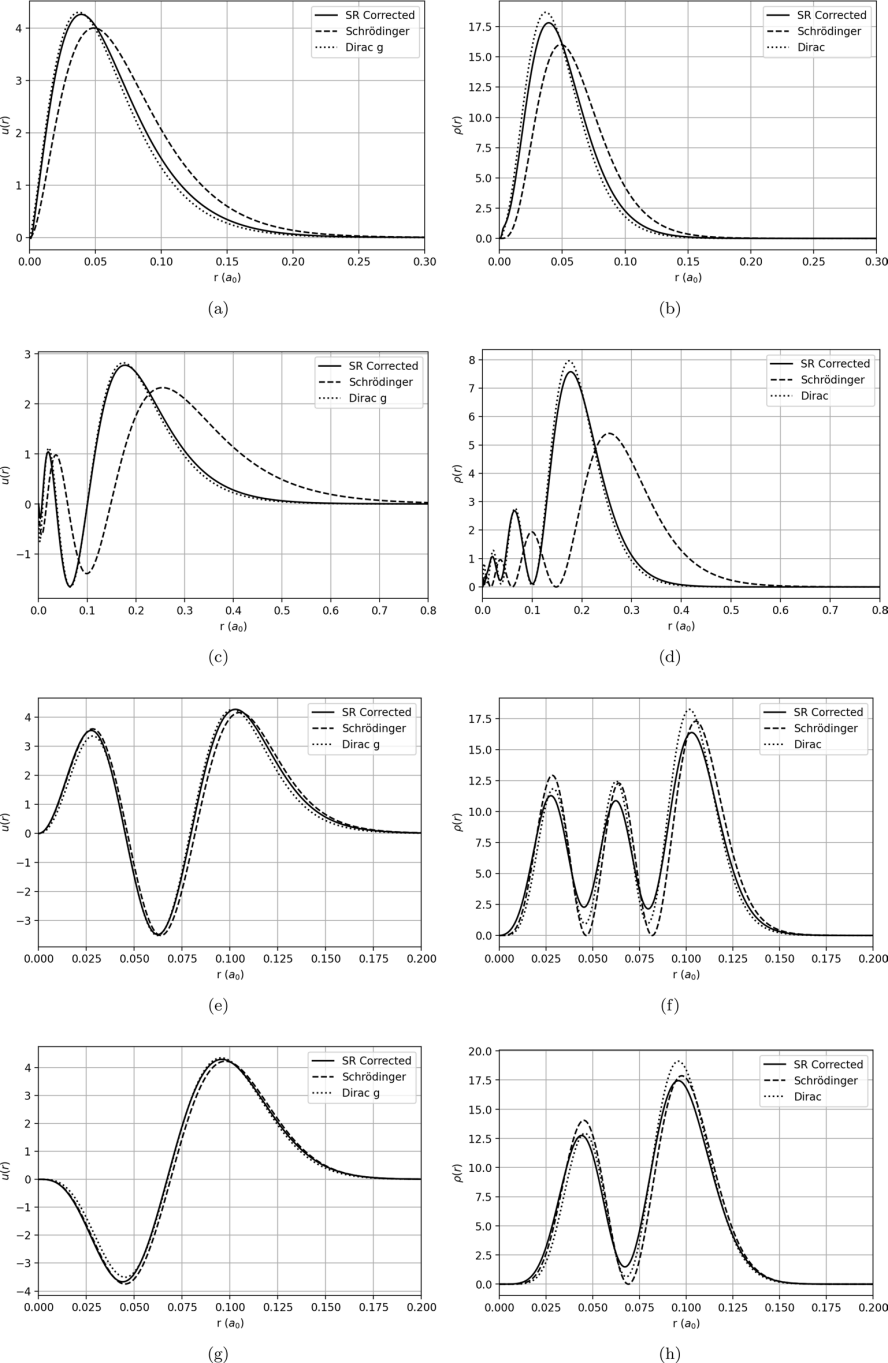
Comparison between selected wavefunctions (left column) and probability densities (right column) across the four studied potentials: (**a**,**b**) Coulomb $2p_{1/2}$; (**c**,**d**) Yukawa $4s_{1/2}$; (**e**,**f**) Woods–Saxon $4p_{3/2}$; (**g**,**h**) Harmonic Oscillator $4d_{5/2}$.

As expected, relativistic corrections are negligible in Hydrogen but become substantial for heavy nuclei such as Lead and superheavy ones like Oganesson. We investigate this by comparing the wavefunctions and probability densities for both modified (as introduced in this work) and standard Schrödinger equations, as well as the large component of the Dirac equation using a series of different potentials. Firstly, we study Lead using a Coulomb potential Lead, treating it as a hydrogenic atom. We also investigate Oganesson using a Yukawa screening potential. For heavier atomic systems, one often considers to introduce effective Coulomb potentials of various form to account for effect from the electron screening, exchange, and self-consistency (see, for example, Ref^46^.). To further investigate our formalism in a more general view, we also study the effects on systems with a Woods-Saxon potential and harmonic oscillator potential. A few selected states of each potential are provided in Fig. 2, and a brief description and discussion on each of the potentials follows in the next subsections.

Regarding the scope and accuracy, one should note the limits of the applicability of this formalism. Firstly, the effective-mass expansion in Equation 60 is valid in the regime $|E-\Delta |\ll m$, and degrades as the binding gets very deep or $|\nabla \Delta |$ grows (i.e., the potential becomes steeper). Furthermore, we note that for s states the relative energy error is slightly higher, decreasing with increasing $\ell$. The accuracy is also dependent on the type of potential. As a rule of thumb, the formalism is quantitatively reliable for moderate-Z and gently varying central fields, and for sharper potential and higher-Z systems, the higher-order terms become increasingly relevant. Even in that regime, the formalism remains qualitatively correct, since we recover the nodal smearing due to Equation 1 even in those limits.

#### Coulomb potential

In order to study the corrections in a Coulomb potential (Equation 91), we use Pb ($Z=82$) as a case study. This is a heavy element for which relativistic corrections are already noticeable for the deeper states. We have solved for this potential using Dirac, Schrödinger and our modified Schrödinger with spin-relativistic corrections, and compared the energies of various states obtained using the three formalisms, which are tabled in the Appendix section. The wavefunction and probability density for a selected state is provide in the top two panels of Fig. 2, for reference. The spin-relativistic-corrected Schrödinger form explored in this work very neatly reproduces the large component of the Dirac equation, showing the effectiveness of the correction terms in bridging the two formalisms.

#### Yukawa potential

The next potential that was studied was a Yukawa potential. It would also be of interest to look at an example of system where corrections would be very significant. Therefore, using this potential, we modelled Oganesson ($Z=118$), the largest superheavy element synthesized as of the development of this work. We consider this to be a particularly relevant case, especially in a time when the study of superheavy elements such as those beyond $Z=114$ and the influence of relativistic effects in their physicochemical structure is an active and interesting field of research aligned with NuPECC’s Long Range Plan for European Nuclear Physics^47^. It is well known that Oganesson is heavy enough that relativistic corrections become extremely important, as well as a series of corrections namely Breit and QED contributions, vacuum polarization, among others^48^. Therefore, the simplistic Coulomb potential approach we used before becomes rather unfitting, and more robust methods are necessary to discuss its electronic shell structure^49^. Nonetheless, recent results seem to indicate that in superheavy elements the single-electron wave function is fairly similar to the bare electron wave function after the screening effect is taken into account^50^. Therefore, for a reasonable order-of-magnitude comparison, we model Oganesson via a Yukawa potential

92 $$\begin{aligned} V(r)=-\frac{e^2}{4\pi \epsilon \hbar c}\frac{Z}{r}e^{-r/a}, \end{aligned}$$

with *a* a Thomas–Fermi screening length given by

93 $$\begin{aligned} a=\frac{0.8853~a_0}{Z^{1/3}}. \end{aligned}$$

The obtained energy levels, compared with those retrieved from Dirac and the standard Schrödinger equation, can be found in the Appendix, in Table 2. Furthermore, the wavefunction and probability density of an illustrative state are given in the second row of Fig. 2. Note that for our Oganesson model, the deep states are highly relativistic, and therefore the wavefunctions are significantly shifted in relation to the Schrödinger solution, as can be seen by the Dirac solution. The formalism derived in this work reproduces the Dirac solution remarkably well. Namely, the non-vanishing behavior of the probability density in the zeroes of the wavefunction, which is ensured by the second term of Equation 1, is remarkably accurate.

### Woods-Saxon potential

In order to investigate the effects of the correction terms in a potential of Woods-Saxon (WS) type, we used the familiar form

94 $$\begin{aligned} V(r)= -\dfrac{V_0}{1+\exp \!\big (\dfrac{r-R}{a}\big )}, \end{aligned}$$

scaled to reproduce energies in the order of $\textrm{keV}$ for ease of comparison with the previous potentials. Naturally, the reproduced energies don’t correspond to electron shells of an atom, since WS is a nuclear potential and cannot reproduce the 1/*r* Coulomb tail, which is much more adequate for atomic purposes. In any case, we consider it to be of academic interest to study the effects of the corrections in this type of potential as well, as it differs only from the nuclear case via a scaling. For this purpose, we used a radius parameter of $R=30\mathrm {~fm/\hbar c}$, diffuseness of $a=5\mathrm {~fm/\hbar c}$ and depth of $V_0=0.2\mathrm {~MeV}$. The energy table can be found in the Appendix, and a comparison between two wavefunctions and probability densities can be found in the third row of Fig. 2.

### Harmonic oscillator

The final type of potential investigated in this work was the Harmonic Oscillator potential. The usual form of the Harmonic Oscillator potential is given by

95 $$\begin{aligned} V(r)=\frac{1}{2}m\omega ^2r^2, \end{aligned}$$

where *m* represents the mass of the particle, $\omega$ is the angular frequency of the oscillator and *r* is the position. A perturbative analysis of this case has been performed in a previous section, and here we shall focus on the numerical implementation. In order to simulate energies in orders of magnitude comparable with the previous potentials analysed in this work, we have taken $\omega =0.02\mathrm {~s^{-1}}$ and assumed an electron trapped in the potential. We then introduced a shift on the potential by $-k$,with $k=0.2\mathrm {~MeV}$. We do this in order to shift the eigen-energies into a depth at which relativistic corrections become appreciable and comparable to the previous potentials. The wavefunction and probability density of a selected state is provided in the fourth row of Fig. 2 and the energies of selected states can be found in the Appendix.

## Conclusions

In this work we have recovered that the non-relativistic reduction of the Dirac equation, when performed for finite scalar and vector potentials without a prior neglecting order-$1/m^{2}$ terms, naturally yields a Schrödinger-type equation augmented by four relativistic operators. Collecting the momentum–dependent pieces into an energy- and potential-dependent effective mass leads to a compact representation, henceforth called the modified Schrödinger equation, that reproduces the conventional Schrödinger dynamics in the $E,\,\Delta \!\ll \! m$ limit, while retaining a series of correction terms that start to become relevant as elements become heavier and the respective electron shells more relativistic.

Numerically, casting the radial equation as a quadratic eigenvalue problem and solving it by sparse-matrix linearization within a finite-difference discretization furnishes a stable and computationally inexpensive scheme. We use this method to compute and compare the Schrödinger, Dirac and spin-relativistic-corrected Schrödinger formalisms in terms of their energies and wavefunctions and observe the effect of the corrections and their effectiveness. The method reproduces hydrogenic spectra very precisely, which is expected for low energy orbits, and also exposes sizeable deviations for inner shells of heavy ions. The analysis is extended to Lead and Oganesson, respectively modelled as Coulomb and Yukawa potentials, as well as Woods-Saxon and Harmonic Oscillator-type potentials.

The practical value of the present formulation is three-fold. First, it offers a didactically transparent path from the Dirac formalism to quantitatively reliable wave-functions in atomic, nuclear and condensed-matter problems where relativistic corrections are important but a full four-component calculation is unwarranted. Second, by exposing the effective-mass structure it connects naturally to semi-relativistic models used in band-structure and envelope-function approaches. Third, the quadratic-eigenvalue discretization and subsequent linearization allows a highly optimized implementation using modern sparse-linear-algebra libraries, enabling large-scale simulations on commodity hardware.

## Supplementary Information

Supplementary Information.

## Data Availability

The uniform- and logarithmic-mesh solvers developed for the numerical implementation section of this work are openly available as a pack on Zenodo, which can be found on https://doi.org/10.5281/zenodo.17023934.

## Appendix A: energy level tables

See Tables 1, 2, 3, and 4.

**Table 1:** Selected bound-state energies for a Coulomb potential with $Z=82$ (Eq. 91). Columns list *n*, $\ell$, *j*, and energies for Schrödinger (Schr.), modified Schrödinger (Mod-Schr., this work), and Dirac (large component).

*n*	$\ell$	*j*	Schr. (eV)	Mod-Schr. (eV)	Dirac (eV)
1	0	1/2	88769	95737	100705
2	0	1/2	22529	25208	27034
1	1	1/2	22871	26860	27234
1	1	3/2	22871	23101	23339
3	0	1/2	10064	11332	11900
2	1	1/2	10165	11562	11973
2	1	3/2	10165	10368	10758
1	2	3/2	10165	10493	10777
1	2	5/2	10165	10263	10245
4	0	1/2	5675	6240	6618
3	1	1/2	5718	6342	6651
3	1	3/2	5718	5833	6131
2	2	3/2	5718	5891	6143
2	2	5/2	5718	5791	5906
1	3	5/2	5718	5795	5912
1	3	7/2	5718	5750	5738

**Table 2:** Selected bound-state energies for a Yukawa potential with $Z=118$ (see Equation 92). Columns list principal quantum number *n*, orbital $\ell$, total *j*, and energies for Schrödinger (Schr.), modified Schrödinger with spin-relativistic corrections (Mod-Schr., this work), and Dirac (large component).

*n*	$\ell$	*j*	Schr. (eV)	Mod-Schr. (eV)	Dirac (eV)
1	0	1/2	164461	182315	229897
2	0	1/2	30905	39891	52958
1	1	1/2	31501	54089	53947
1	1	3/2	31501	33849	33615
3	0	1/2	7679	14808	14773
2	1	1/2	7644	14219	14892
2	1	3/2	7644	8793	9240
1	2	3/2	7080	8266	8717
1	2	5/2	7080	7375	7352
4	0	1/2	1395	3506	3752
3	1	1/2	1280	3218	3673
3	1	3/2	1280	1649	1926
2	2	3/2	912	1273	1548
2	2	5/2	912	1032	1145
1	3	5/2	318	429	549
1	3	7/2	318	359	330

**Table 3:** Selected bound-state energies for a Woods-Saxon potential with $V_0=0.2\mathrm {~MeV}$, $R=30\mathrm {~fm/\hbar c}$, $a=5\mathrm {~fm/\hbar c}$ (see Equation 94).

*n*	$\ell$	*j*	Schr. (eV)	Mod-Schr. (eV)	Dirac (eV)
1	0	1/2	178573	178332	179047
1	1	1/2	161800	162767	163664
1	1	3/2	161800	162373	162536
1	2	3/2	143103	145048	145951
1	2	5/2	143103	144247	144392
2	0	1/2	139558	141779	143293
1	3	5/2	122854	126159	127040
1	3	7/2	122854	124862	125001
2	1	1/2	118036	121523	124335
2	1	3/2	118036	121003	122942
2	2	3/2	95811	101152	104032
2	2	5/2	95811	100208	102213
3	0	1/2	93497	99004	102504
3	1	1/2	70477	78162	82613
3	1	3/2	70477	77301	81096
4	0	1/2	46940	56152	61431

**Table 4:** Selected bound-state energies for a Harmonic Oscillator potential with $\omega =0.02\mathrm {~s^{-1}}$, $k=0.2\mathrm {~MeV}$ (see Equation 95). Columns list principal quantum number *n*, orbital $\ell$, total *j*, and energies for Schrödinger (Schr.), modified Schrödinger with spin-relativistic corrections (Mod-Schr., this work), and Dirac (large component).

*n*	$\ell$	*j*	Schr. (eV)	Mod-Schr. (eV)	Dirac (eV)
1	0	1/2	169939	170305	170454
1	1	1/2	150000	151046	152142
1	1	3/2	150000	151046	150716
2	0	1/2	129909	131166	133393
1	2	3/2	130000	131683	132928
1	2	5/2	130000	131683	131167
2	1	1/2	110001	112912	115611
2	1	3/2	110001	112912	114132
1	3	5/2	110000	112493	113875
1	3	7/2	110000	112493	111799
3	0	1/2	89887	92487	97236
2	2	3/2	90001	94036	96881
2	2	5/2	90001	94036	95043
3	1	1/2	70002	75709	79933
3	1	3/2	70002	75709	78407
4	0	1/2	49869	54021	61901
