# Supplementary material for: Closed-form spin-relativistic corrections from the Dirac equation enabling a modified Schrödinger solver
Source: Sci Rep. 2025 Dec 21;16:94. doi: 10.1038/s41598-025-29243-4 (PMC12765018; doi:10.1038/s41598-025-29243-4)
Supplement: Supplementary file 1 — Supplementary Information. [file 41598_2025_29243_MOESM1_ESM.pdf]

Supplementary Material: Energy Level Tables for ”Closed-Form Spin-Relativistic Corrections from the Dirac Equation Enabling a Modified Schrödinger Solver”

Mário B. Amaro, Nazeef, Camille J. Dussech, and Chong Qi

OVERVIEW

This Supplementary Material provides the appendix tables referenced in the main manuscript. They report bound-state energies for selected states computed using three formalisms: the standard Schrödinger equation ("Schr."), the spin-relativistic-corrected modified Schrödinger equation introduced in this work ("Mod-Schr."), and the large component of the Dirac equation ("Dirac").

TABLE I: Selected bound-state energies for a Coulomb potential with nuclear charge $Z = 82$ (lead). Columns list principal quantum number n, orbital ℓ, total j, and energies for Schrödinger (Schr.), modified Schrödinger (Mod-Schr., this work), and Dirac (large component).

n	ℓ	j	Schr. (eV)	Mod-Schr. (eV)	Dirac (eV)
1	0	1/2	88 769	95 737	100 705
2	0	1/2	22 529	25 208	27 034
1	1	1/2	22 871	26 860	27 234
1	1	3/2	22 871	23 101	23 339
3	0	1/2	10 064	11 332	11 900
2	1	1/2	10 165	11 562	11 973
2	1	3/2	10 165	10 368	10 758
1	2	3/2	10 165	10 493	10 777
1	2	5/2	10 165	10 263	10 245
4	0	1/2	5675	6240	6618
3	1	1/2	5718	6342	6651
3	1	3/2	5718	5833	6131
2	2	3/2	5718	5891	6143

(continued)

n	ℓ	j	Schr. (eV)	Mod-Schr. (eV)	Dirac (eV)
2	2	5/2	5718	5791	5906
1	3	5/2	5718	5795	5912
1	3	7/2	5718	5750	5738

TABLE II: Selected bound-state energies for a Yukawa-screened potential with $Z = 118$ (Oganesson) and Thomas–Fermi screening length $a = 0.8853\, a_0/Z^{1/3}$. Columns list n, ℓ, j, and energies for Schrödinger (Schr.), modified Schrödinger (Mod-Schr.), and Dirac (large component).

n	ℓ	j	Schr. (eV)	Mod-Schr. (eV)	Dirac (eV)
1	0	1/2	164 461	182 315	229 897
2	0	1/2	30 905	39 891	52 958
1	1	1/2	31 501	54 089	53 947
1	1	3/2	31 501	33 849	33 615
3	0	1/2	7679	14 808	14 773
2	1	1/2	7644	14 219	14 892
2	1	3/2	7644	8793	9240
1	2	3/2	7080	8266	8717
1	2	5/2	7080	7375	7352
4	0	1/2	1395	3506	3752
3	1	1/2	1280	3218	3673
3	1	3/2	1280	1649	1926
2	2	3/2	912	1273	1548
2	2	5/2	912	1032	1145

(continued)

n	ℓ	j	Schr. (eV)	Mod-Schr. (eV)	Dirac (eV)
1	3	5/2	318	429	549
1	3	7/2	318	359	330

TABLE III: Selected bound-state energies for a Woods-Saxon potential with depth $V_0 = 0.2\,\text{MeV}$, radius $R = 30\,\text{fm}/\hbar c$, and diffuseness $a = 5\,\text{fm}/\hbar c$. Columns list n, ℓ, j, and energies for Schrödinger (Schr.), modified Schrödinger (Mod-Schr.), and Dirac (large component).

n	ℓ	j	Schr. (eV)	Mod-Schr. (eV)	Dirac (eV)
1	0	1/2	178 573	178 332	179 047
1	1	1/2	161 800	162 767	163 664
1	1	3/2	161 800	162 373	162 536
1	2	3/2	143 103	145 048	145 951
1	2	5/2	143 103	144 247	144 392
2	0	1/2	139 558	141 779	143 293
1	3	5/2	122 854	126 159	127 040
1	3	7/2	122 854	124 862	125 001
2	1	1/2	118 036	121 523	124 335
2	1	3/2	118 036	121 003	122 942
2	2	3/2	95 811	101 152	104 032
2	2	5/2	95 811	100 208	102 213
3	0	1/2	93 497	99 004	102 504
3	1	1/2	70 477	78 162	82 613
3	1	3/2	70 477	77 301	81 096
4	0	1/2	46 940	56 152	61 431

TABLE IV: Selected bound-state energies for a Harmonic Oscillator potential with $\omega = 0.02\,\mathrm{s}^{-1}$ shifted by $-k$ with $k = 0.2\,\mathrm{MeV}$. Columns list n, ℓ, j, and energies for Schrödinger (Schr.), modified Schrödinger (Mod-Schr.), and Dirac (large component).

n	ℓ	j	Schr. (eV)	Mod-Schr. (eV)	Dirac (eV)
1	0	1/2	169 939	170 305	170 454
1	1	1/2	150 000	151 046	152 142
1	1	3/2	150 000	151 046	150 716
2	0	1/2	129 909	131 166	133 393
1	2	3/2	130 000	131 683	132 928
1	2	5/2	130 000	131 683	131 167
2	1	1/2	110 001	112 912	115 611
2	1	3/2	110 001	112 912	114 132
1	3	5/2	110 000	112 493	113 875
1	3	7/2	110 000	112 493	111 799
3	0	1/2	89 887	92 487	97 236
2	2	3/2	90 001	94 036	96 881
2	2	5/2	90 001	94 036	95 043
3	1	1/2	70 002	75 709	79 933
3	1	3/2	70 002	75 709	78 407
4	0	1/2	49 869	54 021	61 901
